# Supplementary material for: Cross-cultural adaptation and psychometric validation of a Spanish version of the Maryland Assessment of Recovery Scale (MARS-12)
Source: PLoS One. 2024 Feb 23;19(2):e0298554. doi: 10.1371/journal.pone.0298554 (PMC10889629; doi:10.1371/journal.pone.0298554)
Supplement: Attachment — Submitted filename: Response to reviewers.docx [file pone.0298554.s001.docx]

Dear Academic Editor,

First of all, we would like to express our gratitude for the reviews received. We believe that the reviewers´ suggestions have allowed us to improve the quality of our manuscript. All changes in the revised manuscript are marked with track changes in the paper we have submitted via the online submission system. In the next few lines, we respond to the Journal requirements, to additional editor’s comments and the reviewers’ comments one by one.

**RESPONSE TO JOURNAL REQUIREMENTS:**

**When submitting your revision, we need you to address these additional requirements.**

**1. Please ensure that your manuscript meets PLOS ONE's style requirements, including those for file naming.**

We have ensured that the manuscript meets PLOS ONE’s style requirements, including those for file naming.

**2. Note from Emily Chenette, Editor in Chief of PLOS ONE, and Iain Hrynaszkiewicz, Director of Open Research Solutions at PLOS:**

**Did you know that depositing data in a repository is associated with up to a 25% citation advantage (https://doi.org/10.1371/journal.pone.0230416)? If you’ve not already done so, consider depositing your raw data in a repository to ensure your work is read, appreciated and cited by the largest possible audience. You’ll also earn an Accessible Data icon on your published paper if you deposit your data in any participating repository (**[**https://plos.org/open-science/open-data/#accessible-data**](https://plos.org/open-science/open-data/#accessible-data)**).**

We have deposited data in the OSF: DOI 10.17605/OSF.IO/8U5YF

**3. When completing the data availability statement of the submission form, you indicated that you will make your data available on acceptance. We strongly recommend all authors decide on a data sharing plan before acceptance, as the process can be lengthy and hold up publication timelines. Please note that, though access restrictions are acceptable now, your entire data will need to be made freely accessible if your manuscript is accepted for publication. This policy applies to all data except where public deposition would breach compliance with the protocol approved by your research ethics board. If you are unable to adhere to our open data policy, please kindly revise your statement to explain your reasoning and we will seek the editor's input on an exemption. Please be assured that, once you have provided your new statement, the assessment of your exemption will not hold up the peer review process.**

We have deposited the data in the OSF: DOI 10.17605/OSF.IO/8U5YF

**4. We note that you have referenced (ie. Optum. Maryland Assessment of Recovery Scale — MARS-12 [Internet]) which has currently not yet been accepted for publication. Please remove this from your References and amend this to state in the body of your manuscript: (ie “Bewick et al. [Unpublished]”) as detailed online in our guide for authors.**

We have removed the unpublished reference and amend it in the body of the manuscript.

**RESPONSE TO ADDITIONAL EDITOR COMMENTS:**

- **The study presents original research.**
- **The authors should clearly highlight the new contributions that MARS-12 adds to the practice.**

We have clearly highlighted the contributions that MARS-12 adds to the practice (pages 17 (lines 356-363) and 18 (lines 381-385) in the track changes version).

- **Revise the state of the art (with more recent evidence), discussion, and conclusions as sugested.**

We have added new ideas based on recent evidence both in the introduction and in the discussion. In fact, we have added 17 new references.

**RESPONSE TO REVIEWER #1:**

**Very interesting topic for the practice of Mental Health and Psychiatric Nursing.**

**In general, it is well structured, complying with the guidelines for the adoption and validation of a scale.**

**The research took into account credible scientific databases and other complementary literature.**

We are very grateful for your kind words.

**RESPONSE TO REVIEWER #2:**

**Thanks you for your article titled "Cross-cultural Adaptation and Psychometric Validation of a Spanish Version of the Maryland Assessment of Recovery Scale (MARS-12)." Overall, this study has been conducted appropriately, meeting all the criteria required for research of this nature.**

**The article is well-prepared for publication, with a clear structure, comprehensive methodology, and relevant results. The topic is timely and significant. However, it is crucial to highlight the benefits of this instrument for care teams, users, and their families. Subsequently, strengthening the discussion of the data and presenting the study's conclusion would be beneficial.**

**My main concern relates to the literature review, with 70% of the documents used being over 5 years old.**

**Specific Comments:**

**Abstract:**

- **The abstract should clearly outline the method used in this study, and it would be helpful to specify the contexts to which the 325 participants belong.**

Following the reviewer’s suggestion, he have included in the abstract information on the method used in the study and the context to which the participants belong.

**Introduction:**

- **Consider expanding the literature review in the introduction, focusing on current research on self-care management and self-monitoring Clarify the benefits of the instrument for professionals, users, and families.**

We have expanded the literature review in the introduction, focusing on current research on the recovery-based approach to mental health care which emphasizes self-care management and self-monitoring. We have also added a last paragraph highlighting the practical implications of this study and adding some ideas about the benefits of the instrument to promote self-determination and self-management in the recovery process.

We have added new ideas and 17 recent references in order to reflect the current conceptualization of the recovery-oriented perspective.

Furthermore, we have clarified, in the discussion (page 16) the benefits of the instrument for professionals, users and families.

**Materials and Methods:**

- **The materials and methods section appears to be adequate and well-explored, according to your feedback.**

**Results:**

- **Confirm that the results are presented clearly and concisely.**

**Discussions (lines 275-312):**

- **The discussion section deserves further exploration. Include references from authoritative authors regarding the scale's evaluated items and their benefits.**

We have added a reference to a systematic review focused on personal recovery measures and we have used it as a basis for pointing out the advantages of the MARS-12 over previously developed long versions of the scale.

- **Make comparisons with recent studies on the topic, using more up-to-date bibliography.**

We have answered to this suggestion adding more recent studies on the topic in the introduction and in the discussion.

- **Evaluate whether the current discussion is sufficient for such a relevant and pertinent theme and if it adequately represents the work done in this study.**

**Conclusion:**

- **Clearly identify the conclusions drawn from the study.**

In the last paragraph of the discussion of the original version of the manuscript some conclusions derived from the study were already summarized. However, following the suggestion of the reviewer we have added a last paragraph focusing mainly on the practical implications of this work so that they can be clearly identified.

**References:**

- **Note that references, on the whole, are over 5 years old and suggest improvements by incorporating more recent sources.**

We have incorporated 17 more recent references throughout the study and we have removed 7 older ones.

We look forward to hearing from you in due time regarding our submission and to respond to any further questions and comments you may have.

Sincerely,

Jone Aliri
